# Spatial detection of fetal marker genes expressed at low level in adult human heart tissue

**DOI:** 10.1038/s41598-017-13462-5

**Published:** 2017-10-11

**Authors:** Michaela Asp, Fredrik Salmén, Patrik L. Ståhl, Sanja Vickovic, Ulrika Felldin, Marie Löfling, José Fernandez Navarro, Jonas Maaskola, Maria J. Eriksson, Bengt Persson, Matthias Corbascio, Hans Persson, Cecilia Linde, Joakim Lundeberg

**Affiliations:** 1 Division of Gene Technology, KTH Royal Institute of Technology, Science for Life Laboratory, Stockholm, Sweden; 2 Department of Cell and Molecular Biology, Karolinska Institutet, Stockholm, Sweden; 3 Department of Molecular Medicine and Surgery, Karolinska Institutet, Stockholm, Sweden; 4 Department of Clinical Physiology, Karolinska University Hospital, Stockholm, Sweden; 5 Department of Molecular Biology, Uppsala University, Science for Life Laboratory, Uppsala, Sweden; 6 Department of Medical Biochemistry and Biophysics, Karolinska Institutet, Science for Life Laboratory, Stockholm, Sweden; 7 Department of Cardiothoracic Surgery and Anesthesiology, Karolinska University Hospital, Solna, Sweden; 8 Department of Cardiology, Danderyd Hospital, Stockholm, Sweden; 9 Department of Clinical Sciences, Danderyd Hospital, Karolinska Institutet, Stockholm, Sweden; 10 Department of Medicine, Karolinska Institutet, Stockholm, Sweden; 11 Department of Cardiology, Karolinska University Hospital, Stockholm, Sweden

## Abstract

Heart failure is a major health problem linked to poor quality of life and high mortality rates. Hence, novel biomarkers, such as fetal marker genes with low expression levels, could potentially differentiate disease states in order to improve therapy. In many studies on heart failure, cardiac biopsies have been analyzed as uniform pieces of tissue with bulk techniques, but this homogenization approach can mask medically relevant phenotypes occurring only in isolated parts of the tissue. This study examines such spatial variations within and between regions of cardiac biopsies. In contrast to standard RNA sequencing, this approach provides a spatially resolved transcriptome- and tissue-wide perspective of the adult human heart, and enables detection of fetal marker genes expressed by minor subpopulations of cells within the tissue. Analysis of patients with heart failure, with preserved ejection fraction, demonstrated spatially divergent expression of fetal genes in cardiac biopsies.

## Introduction

Heart failure (HF) is one of the leading health problems with more than 23 million cases worldwide. In this syndrome the heart is unable to provide the body with a sufficient blood supply to maintain homeostasis, and is being characterized as an emerging epidemic^1^. Thus, there have been numerous studies of failing adult heart tissue during the past decade, using various molecular approaches such as microarray analyses, quantitative real-time PCR, and RNA sequencing (RNA-seq)^2–6^. Detailed RNA-seq studies of healthy and failing human myocardium have revealed remarkable similarity between upregulated genes in the failing heart and fetal myocardium^5^. Considering this, fetal marker gene expression could potentially be responsible for the remodeling of the failing human heart.

Two complicating factors when analyzing adult human heart tissue (compared to other human tissue types) is that it contains a large proportion of fibrous tissue and has a low cell density, so disrupting the cells and extracting their total RNA is challenging. Thus, rigorous precautions must be taken to avoid degradation of RNA during its extraction, and (hence) impairment of both RNA quality and yields. A further complication is that in standard RNA-seq, whole tissue biopsies are homogenized and average representations of expression profiles within the entire sample are obtained. Consequently, information on spatial patterns of gene expression is lost and signals from subpopulations of cells with deviant profiles, such as those with low-level fetal marker gene expression, are obscured. To overcome these deficiencies, this study employed the spatial transcriptomics (ST) technology^7^, which enables spatial analysis of fetal marker genes expressed at low levels within whole adult ventricle and atrial tissue sections.

## Results

**Figure 1:**
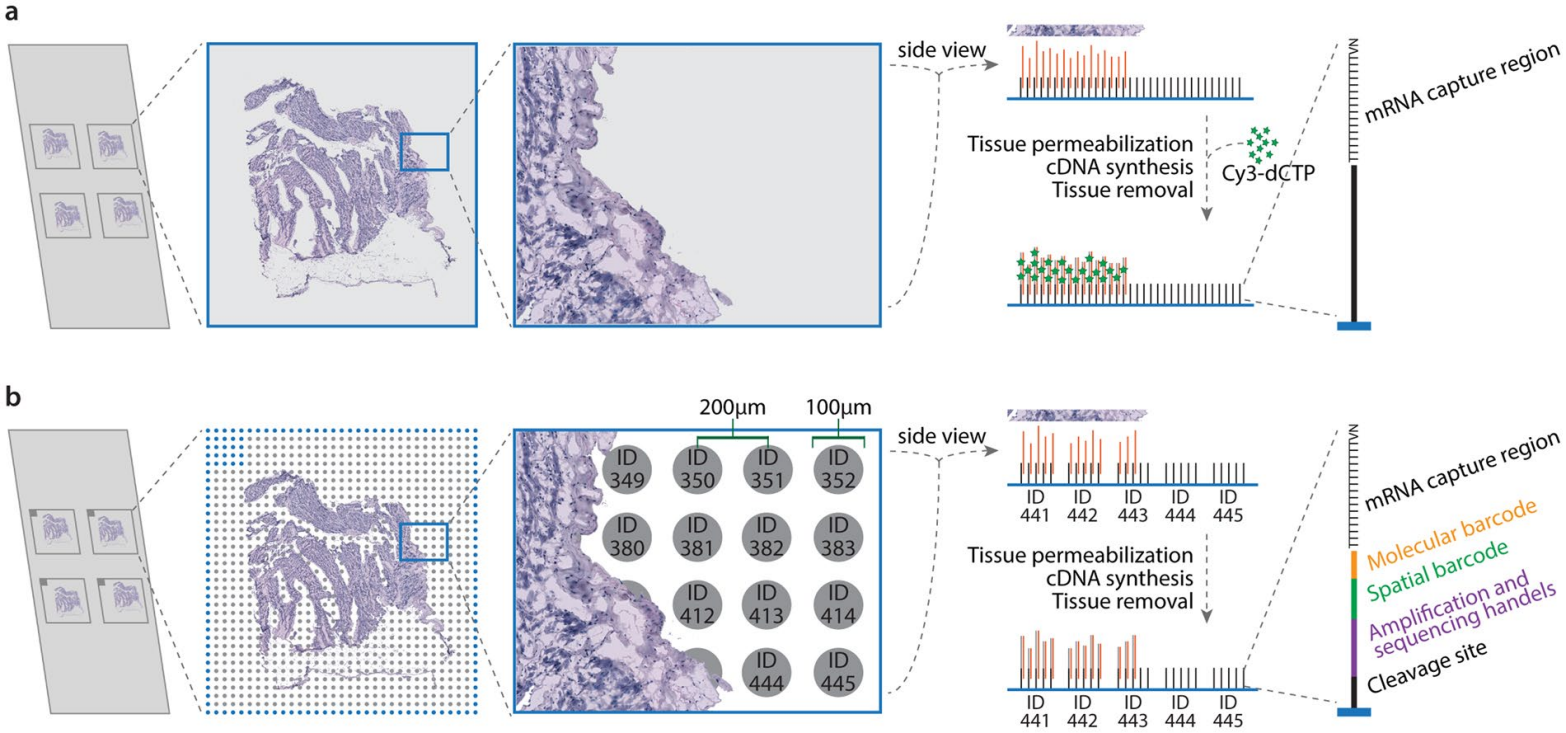
Schematic of spatially resolved gene expression analysis on adult heart. (**a**) In order to establish optimal conditions for retrieval and attachment of mRNA *in situ*, a quality control assay is first used. Intensity of signals generated from the Cy3-cDNA footprint is then scored to examine optimal permeabilization conditions. (**b**) A spatially barcoded microarray, printed with 1,007 clusters (i.e features) of reverse-transcription oligo-dT primers, are then used with established permebilization conditions. Each feature on the array contains more than 200 million probes, all sharing a unique DNA sequence (barcode) specific to that feature. The barcode is used in the downstream analysis to link each feature’s position within the tissue to the mRNA captured at that position. Finally, following reverse transcription and tissue removal, barcoded cDNA is enzymatically released from the array and used to generate sequencing libraries.

In this study right atrial appendage (RAA) and left ventricular needle (LV) biopsies from three male subjects were used (see Table 1 for the subjects’ ages and numbers of samples). First, optimizations of the ST protocol were done by placing thin cardiac tissue sections on a microarray uniformly coated with reverse-transcription oligo-dT primers. The tissue sections were then subjected to permeabilization and reverse transcription (with incorporation of Cy3-labeled nucleotides), directly on the surface, and subsequently removed to score the intensity of signals generated from the Cy3-cDNA footprint (Fig. 1a). Optimizations of fibrous heart tissue were done for both LV (Supplementary Fig. 1) and RAA biopsies (Supplementary Fig. 2). The optimized protocols were thereafter applied to experiments on a spatially barcoded microarray to facilitate identification of specific parts of the investigated tissue (Fig. 1b). The number of cells located within the border of each feature position (i.e. circular area with a diameter of 100 µm) was estimated between five to fifteen for RAA and LV depending on if cardiomyocytes were longitudinal- or cross-sectioned (Supplementary Fig. 3). Longitudinally oriented cardiomyocytes can potentially cover more than one single feature and numerous features contain different cell types such as cardiomyocytes, cardiac fibroblasts and adipocytes. Hence, individual transcriptomes received from each feature will provide spatial gene expression profiles.

**Table 1:** Overview of subjects and samples.

Subject	Left ventricle		Right atrial appendage		Gender/Age
	Adjacent tissue sections	Number of features covered by tissue	Adjacent tissue sections	Number of features covered by tissue	
Individual 1	A	61	C	282	Male
	B	60	D	292	74 years
Individual 2	E	55	N/A*	N/A	Male
	F	52			77 years
Individual 3	G	107	I	534	Male
	H	85	J	365	58 years

*The RAA biopsy from individual 2 contained almost only adipose tissue and generated too little data to be compared with the other samples.

**Figure 2:**
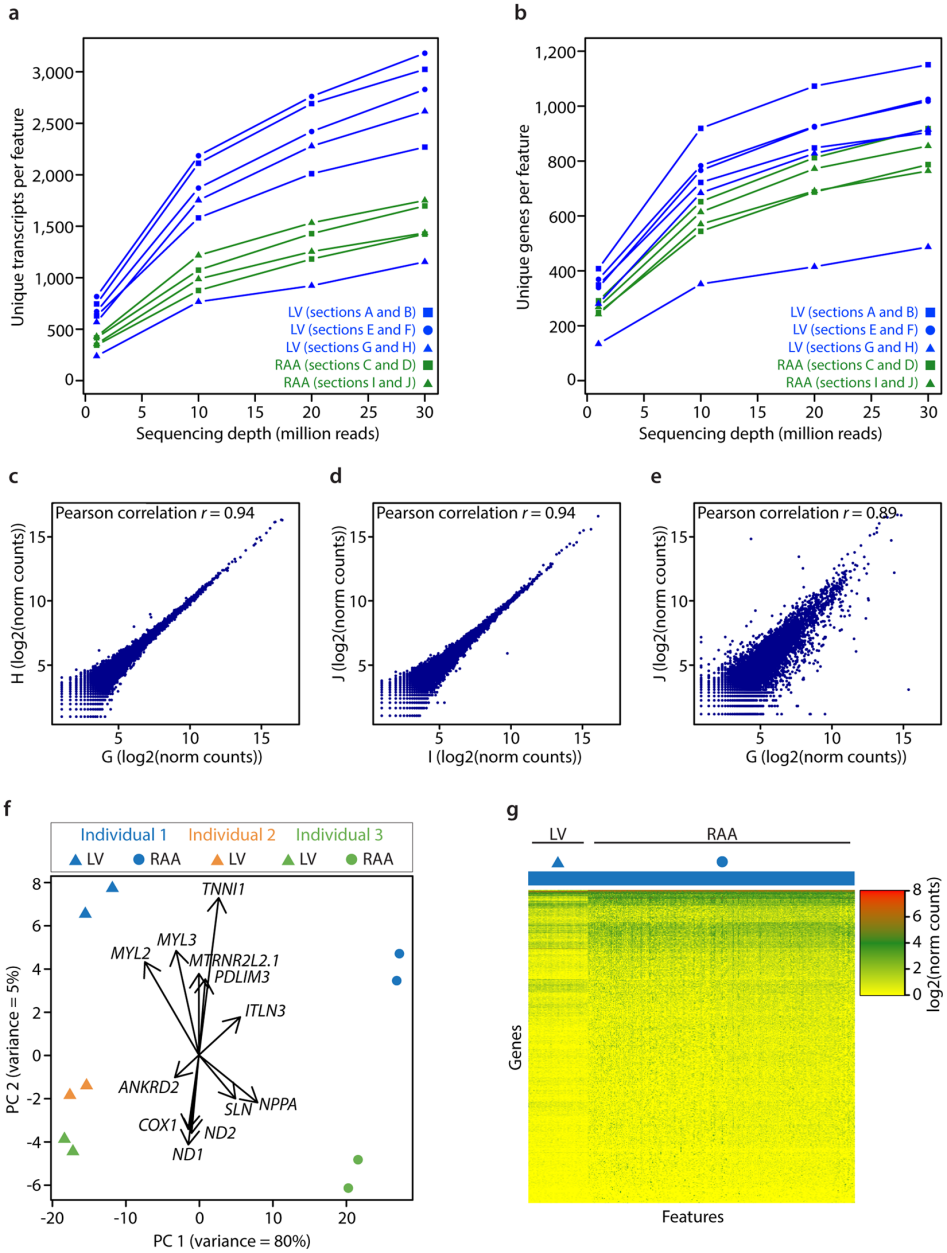
Gene expression patterns within and between individuals. (**a**,**b**) Saturation curves showing numbers of unique transcripts (**a**) and unique genes (**b**) per feature, against subsets of raw reads. (**c** and **d**) Scatterplots showing Pearson correlation of gene expression between consecutive tissue sections from LV and RAA. (**e**) Scatterplot showing Pearson correlation of gene expression between different cardiac regions of the same individual. Letters correspond to tissue sections specified in Table 1. (**f**) Principal Component Analysis score plot showing separation of LV and RAA sections along PC1, and separation of individuals along PC2. Loadings show genes that make a major contribution to the separation. (**g**) Heatmap of gene expression across all features from one LV and one RAA tissue section from individual 1.

All samples were snap frozen upon retrieval and contained RNA of good quality (RIN 7.4–8.8) (Supplementary Fig. 4), but the LV samples yielded on average more data per spatial feature than the RAA samples (Fig. 2a,b), most probably due to their less fibrous property compared to RAA.

All experiments were performed using consecutive sections, as replicates, and pairwise comparisons were done. Consecutive sections from the same sample type, LV or RAA, yielded a strong correlation in gene expression. However, correlations between LV and RAA samples were weaker, as expected (Fig. 2c–e). Further, a principal component analysis (PCA) of all samples showed the same separation of LV and RAA samples, and allowed the identification of marker genes responsible for the separation (Fig. 2f). Marker genes found responsible for the separation between RAA (NPPA, ITLN3 and SLN) and LV (MYL2, MYL3 and ANKRD2) samples in this study were consistent with previously reported findings by the Genotype-Tissue Expression (GTEx) project^8^. The fetal TNNI1 gene substantially contributed to the separation of individual 1, and the mitochondrial genes ND1, ND2 and COX1 contributed to the separation of individual 3. As the variance between cardiac regions (80%) is substantially higher than between individuals (5%), the observed differences between samples are mainly due to the regions being analyzed. The increased number of differentially expressed (DE) genes found between individuals compared to cardiac regions further supports this (Supplementary Fig. 5). To evaluate the extent of differences in spatial gene expression within the RAA and LV tissue sections from the same individual, 1,000 genes with the highest variance across features were investigated. The results revealed some differences in gene expression within the individual tissue sections, although the variation was strongest between the two regions of the adult heart (Fig. 2g).

**Figure 3:**
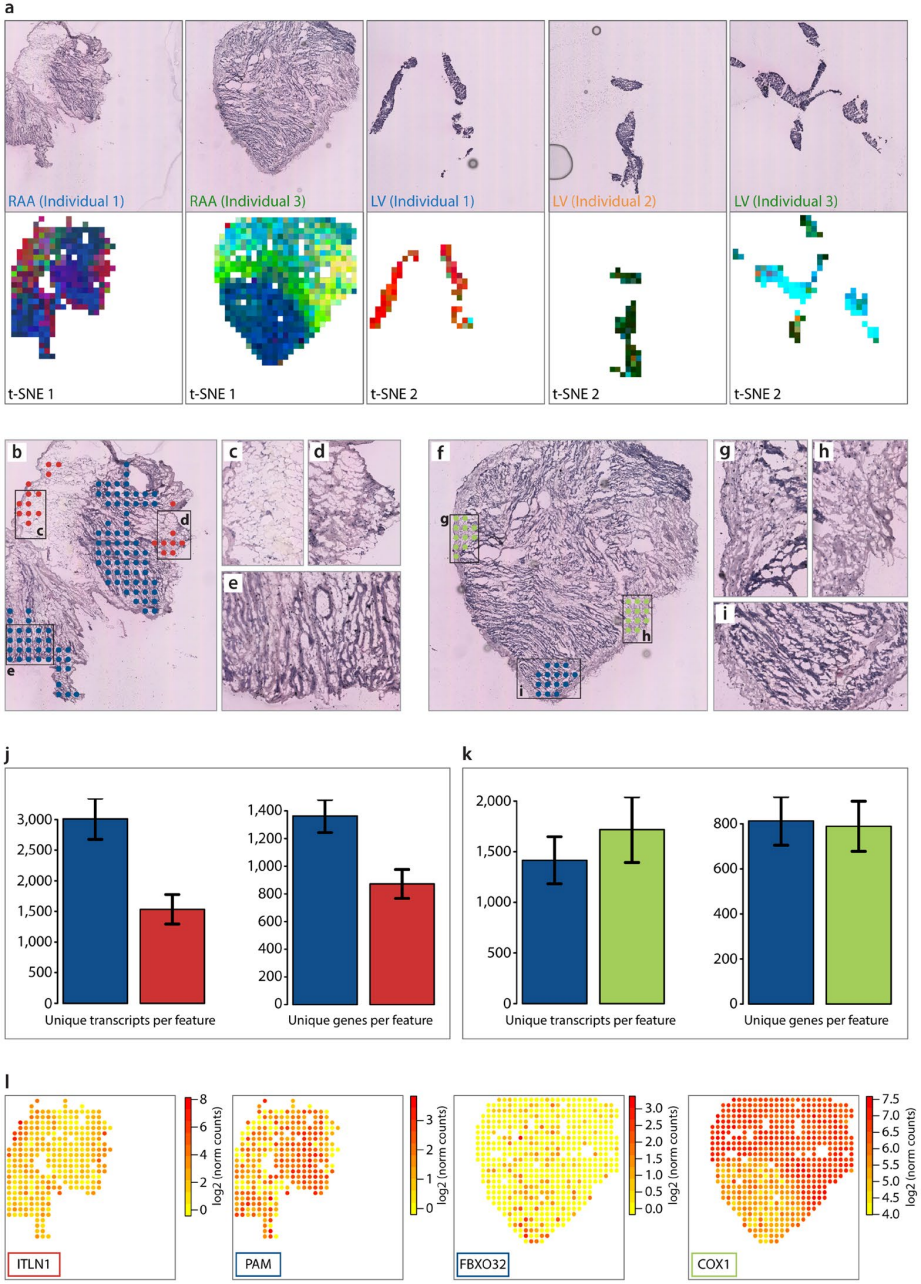
Spatially resolved gene expression patterns within cardiac tissue sections. (**a**) Upper panel: Images of one hematoxylin- and eosin-stained replicate of each sample. Images have been cropped to match the area of the spatially barcoded microarray. Lower panel: Results of applying *t*-SNE analysis to data from RAA (4 samples in total; *t*-SNE 1) and LV (6 samples in total; *t*-SNE 2). Similar colors within the *t*-SNE plots reflect similar gene expression patterns. (**b**–**i**) Cell composition of right atrial appendage. (**b**) Selected features within the RAA of individual 1 corresponding to the colored areas in results of the *t*-SNE 1 analysis shown in (**a**). (**c**, **d** and **e**) Magnified images of corresponding areas in (**b**), showing: (**c**) an adipocyte-tissue-rich region, (**d**) a fibrous-tissue-rich region, and (**e**) a cardiomyocyte-rich region. (**f**) Selected features within the RAA of individual 3 corresponding to the colored areas in results of the *t*-SNE 2 analysis shown in (**a**). (**g**,**h** and **i**) Magnified images of corresponding areas in (**f**), showing: a pericardial fibrous-tissue-rich region (**g**,**h**) and a cardiomyocyte-rich region (**i**). (**j**) Barplots showing that the blue area (corresponding to the cardiomyocyte-rich region) contains ~2-fold more unique transcripts and ~1.6-fold more unique genes than the red area (corresponding to adipocyte- and fibrous-rich tissue). (**k**) Barplots showing that pericardial fibrous-tissue contains similar numbers of unique transcripts and genes per feature to cardiomyocyte-rich regions. Error bars indicate standard errors. (**l**) Spatially resolved expression heatmaps showing four differentially expressed genes between the blue and red area of individual 1, and between the blue and green area of individual 3.

Next, we wanted to investigate each tissue region in depth and visualize potential spatial patterns of gene expression by dimensionality reduction. Analysis with *t*-Distributed Stochastic Neighbor Embedding (*t*-SNE) revealed substantially greater within-tissue variation in gene expression patterns in RAA than in LV (Fig. 3a). As similar colors in the *t*-SNE plots reflect similar gene expression patterns, sets of features with uniform color in RAA samples were examined in more detail. Figure 3b–i shows that the differences in gene expression patterns are associated with the cell composition of the tissue in these feature sets (predominantly cardiomyocytes or regions rich in pericardial-, fibrous- or adipose-tissue). Histological staining was performed in order to confirm annotations of the cell type composition (Supplementary Fig. 6). Average numbers of unique transcripts and genes differ more between cardiomyocyte- and adipocyte/fibrous-rich regions than between cardiomyocyte- and pericardial-rich regions (Fig. 3j,k). Results of subsequent differential gene expression analysis between the colored regions were consistent with the *t*-SNE pattern (Supplementary Fig. 7), as shown by the spatial distribution of four DE genes (ITLN1, PAM, FBXO32 and COX1) in individuals 1 and 3 (Fig. 3l). All four genes have earlier been reported to have selective expression in human epicardial adipose tissue (ITLN1^9^), human cardiac muscle (PAM^10^ and FBXO32^11^) and human endocardium and fibroblasts (COX1^12^), in accordance to this study. No DE genes across the epicardial to endocardial axis of LV samples were recorded.

**Figure 4:**
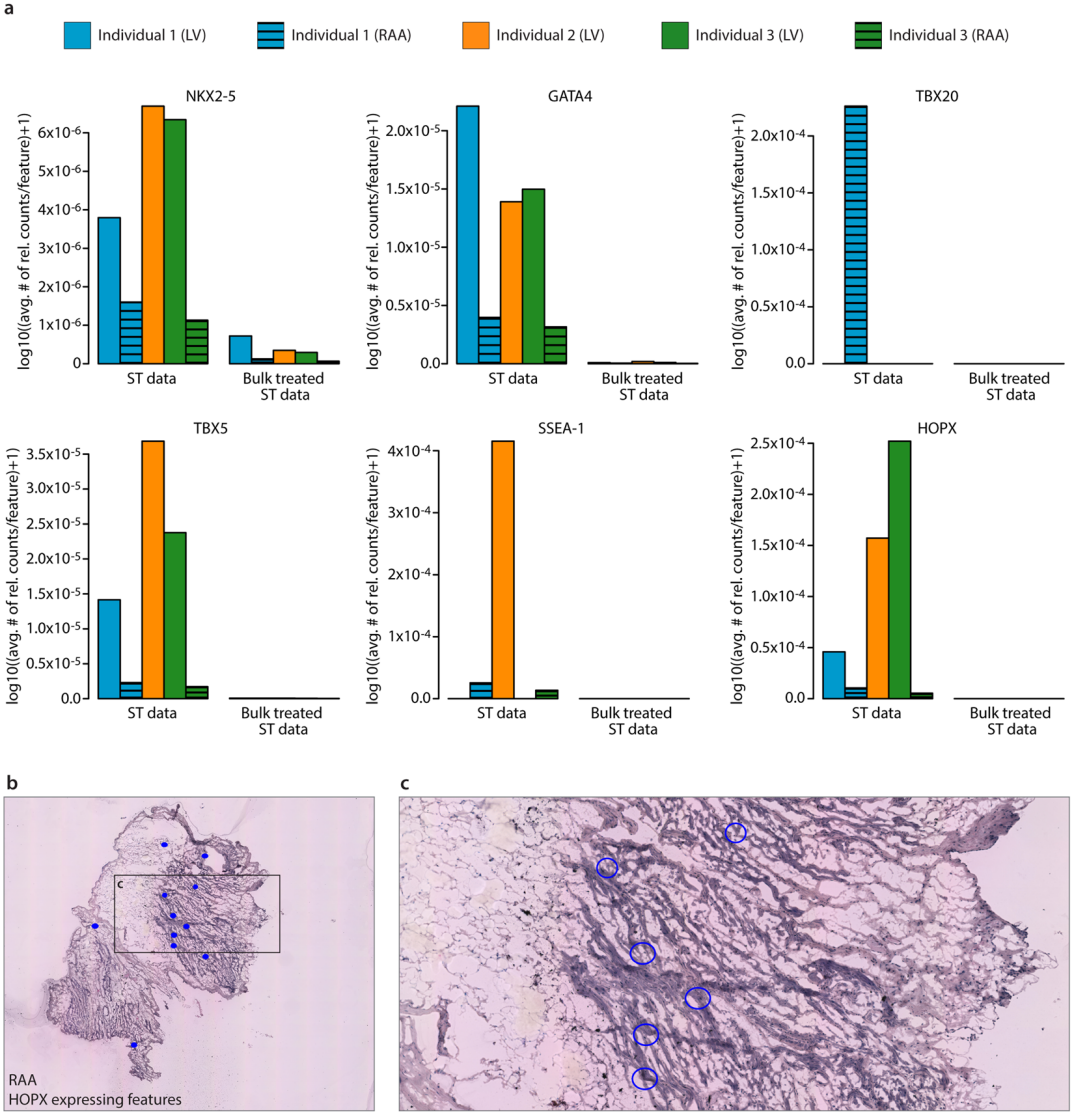
Detection of fetal gene expression within adult heart tissue sections. (**a**) Barplots showing the average number of relative fetal gene counts per feature. All numbers have been log-transformed after adding a pseudocount of 1. In the ST data, the number of features is based on features displaying read counts from indicated fetal marker gene. In the bulk treated ST data, the number of features is based on the total amount of features within the dataset. (**b**) Spatial view of individual features within RAA of individual 1 displaying expression of the HOPX gene. (**c**) Magnified image of corresponding area in (**b**) illustrating an example of features expressing HOPX.

Finally, with confirmed spatial variation in our data, we wanted to investigate the occurrence and position of potential clinical biomarkers of HF, such as fetal genes. The expression of five well-known fetal marker genes (NKX2-5, GATA4, TBX20, TBX5 and SSEA-1), and the recently reported HOPX gene^13^, in the adult heart were investigated. Here, a comparison between ST data and bulk treated ST data was made in order to evaluate the sensitivity of fetal gene detection. Results from this analysis showed that lowly expressed fetal marker genes generate higher detection signals from the ST data than from the bulk data (Fig. 4a). The signal was based on the average amount of relative fetal gene read counts per feature, where the number of features corresponds to features displaying fetal gene read counts (ST data) or to the total amount of features within the dataset (bulk treated ST data) (Supplementary Tables 1–2). Number of unique fetal gene read counts per feature is shown in Supplementary Fig. 8. Notably, individual 1 expressed all six investigated fetal marker genes, and as an example, the HOPX gene is associated mainly with cardiomyocyte-rich areas (Fig. 4b,c). In contrast, individual 2 and 3 displayed no signal of TBX20 gene expression. The same comparison was done for the fetal TNNI1 gene (mentioned above) and the highly expressed adult TNNI3 gene. Though TNNI1 expression differences between individuals could be detected by bulk data and PCA, Supplementary Fig. 9 and Supplementary Tables 3–4 show that TNNI1 is a weakly expressed gene in individual 2 and 3 and thus detected with higher sensitivity with the ST approach. Here TNNI1 expression is compared next to the highly expressed TNNI3 where no difference between ST and bulk data is noted. Thus, with spatial resolution of RNA-seq data from heart tissue, we are able to detect fetal marker genes expressed at low levels potentially involved in the outcome of HF.

## Discussion

ST technology was recently shown to provide an excellent approach for analyzing spatial gene expression patterns in tissue sections from mouse brain and human breast cancer^7^, with independent validation using laser capture microdissection, and single-molecule fluorescence *in situ* hybridization (smFISH). In addition, complete transcriptome benchmarking was also achieved through comparing with the *in situ* hybridization based Allen Brain Atlas. Here we show that this novel technology is applicable to human fibrous heart tissues, and that fetal marker genes expressed at low levels do not risk the fact of being diluted as noise as in bulk data.

Several studies have highlighted the potential value of fetal genes as clinical biomarkers during HF pathogenesis^14–16^, since these genes may be reactivated by the “fetal gene program”. What triggers the reactivation is poorly understood but it may induce apoptosis resistance in cardiomyocytes and thus be highly protective for these cells^17^. Identification of weakly expressed fetal marker genes in thin cardiac tissue sections could therefore be of major importance in clinical settings. Typically, extracting RNA of acceptable quality and yields from fibrous heart bulk samples is known to be challenging, but this study shows that ST technology adapted for adult heart tissue has a capacity for detecting low-level fetal marker gene expression with more sensitivity than is achievable through bulk data. For that purpose, analyzing adult heart biopsies with ST technology has a major advantage compared to standard bulk analysis since it is able to investigate individual transcriptomes of smaller parts within the tissue.

Furthermore, signals from all six investigated fetal genes were found in analyses of individual 1, while five of them were found in individual 2 and 3. Fetal genes also appear to be enriched in the LV compared to RAA tissue (Fig. 4a). In addition, results also show that the TNNI1 gene is the major marker gene driving the separation of individual 1 from the other individuals (Fig. 2f). TNNI1 is not implicated as a gene induced in the fetal gene program, while the fetal form of the adult TNNI3 gene is expressed during early development of the human heart. It has been postulated that there is no reactivation of the fetal TNNI1 gene during the progression of HF^18^, however this study shows that individual 1 had high expression of TNNI1 in both the LV and in the RAA (Supplementary Fig. 5c,d). If reactivation of the fetal TNNI1 gene occurs in a disease state, it may also explain the observed downregulation of mitochondrial genes in individual 1 (Fig. 2f). Mitochondria play a crucial role in the development and progression of HF, and their dysfunction can contribute to reductions in energy production, which can subsequently increase cell mortality rates. As mitochondrial biogenesis is reportedly retarded in early stages of pathological conditions and enhanced during the end-stage^19^, these findings could be indicative of an early response to HF.

However, as this study contains a limited number of subjects of the ongoing PREFERS epidemiological regional study in Stockholm^20^ sample collection (biopsies from 100 patients and peripheral blood from 2000 heart failure patients), no conclusions about HF disease progression can be made. This pilot technical study constitutes three patients, represented by a total of ten tissue sections and a total sample collection of 1893 tissue domains (microarray features corresponding to equal number of individual micro-dissections) (Table 1), sufficient to estimate technical reducibility. Though, this pilot study was required in order to understand tissue handling, permeabilization and the sensitivity of the method before applying it to future larger clinical studies, including a larger human sample size, yielding the statistical power required for conclusions about medically relevant phenotypes. Yet, here we show the advantage of using a spatially resolved technology over bulk RNA-seq for detection of potential biomarkers expressed at low levels, allowing additional insights into the relationship between expression profiles and HF disease progression.

## Materials and Methods

### Ethical Statements

The adult human cardiac biopsies in this study were retrieved from a prospective cohort study^20^ in which all patients had angina pectoris with or without a previous myocardial infarction and were undergoing elective coronary artery bypass surgery. Subjects included in this study are participating with an informed consent. All procedures met ethical stipulations of the Helsinki Convention (Dnr: 2013/1869-31/1, Matthias Corbascio) after approval of the Regional Ethical Review Board (REPN) Stockholm, Sweden. All experiments were performed in accordance with relevant guidelines and regulations.

### Subjects included in the study

All three patients selected for analyses were well characterized and had for HFpEF (Heart failure with preserved ejection fraction) a typical phenotype based on transthoracic echocardiographic examination. The left ventricular (LV) size was normal with end-diastolic diameter ranging from 42 to 48 mm. LV hypertrophy was present in all patients with interventricular septal thickness of 13–20 mm and posterior wall thickness of 9–11 mm. LV ejection fraction, according to the biplane Simpson method, ranged from 57–79%, with no regional wall motion abnormalities. LV global longitudinal strain, a measure of systolic LV deformation was within normal range from −18 to −19,2%. Left atrium (LA) was dilated with LA volume indexed for body surface area exceeding 40 ml/m^2^. A diastolic variable E/é, used for estimation LV filling pressure, ranged from 10–13. Pulmonary artery pressure, estimated from echocardiography data, ranged from 30 to 49 mmHg, and all three patients had a mild tricuspid regurgitation.

### Preparation of quality control arrays and spatially barcoded arrays

For quality control experiments, poly-T_20_VN oligonucleotides (IDT) were uniformly spread onto Codelink-activated microscope glass slides, according to the manufacturer’s instructions. For spatial experiments, 1,007 unique barcoded oligonucleotides with poly-T_20_VN capture regions (IDT) were printed as 1,007 single spots within 6,200 × 6,600 μm areas of the Codelink-activated microscope glass slides. A border of frame oligonucleotides (Eurofins) was printed in the form of 148 single spots around the barcoded oligonucleotide spots in order to retain the orientation. The diameter and spacing (center-to-center) of the printed spots were 100 and 200 μm, respectively^21^.

### Collection and preparation of human adult cardiac biopsies

Adult human cardiac biopsies from the right atrial appendage and needle biopsies from the left and right ventricles were fresh-frozen and embedded in Tissue-Tek (OCT) at the site of surgery. Cryosections of these biopsies, 5 and 10 μm thick respectively, were taken and placed on the Codelink-activated microscope glass slides, which were incubated at 37 °C for 1 minute, fixed in 36.5–38.0% formaldehyde (#F8775, Sigma-Aldrich) diluted 1:10 in 1xPBS (#09-9400, Medicago) for 10 minutes and then washed in 1xPBS.

### Histological staining and imaging

Hematoxylin and eosin: Tissue sections were incubated in Mayer’s Hematoxylin (#S3309, Daco) for 7 minutes, washed in MQ RNase/DNase-free water, incubated in Bluing buffer (#CS702, Daco) for 2 minutes and washed in MQ RNase/DNase-free water again. They were then stained by exposure to eosin (#HT110216) at 1:20 dilution in 0.45 M Tris and 0.5 M acetic acid (pH 6.0), at room temperature until dry followed by incubation at 37 °C for 5 minutes.

Oil Red O: Adipose tissue was visualized by the accumulation of neutral lipids in fat vacuoles, stained with oil red O (Sigma-Aldrich). Tissue sections were fixed in 4% formaldehyde, rinsed in 60% isopropanol and stained with oil red O for 45 minutes.

Picro-Sirius red: Picro-Sirius red staining was performed using Sirius red (0.1%, Histolab) in saturated picric acid according to standard protocols. Fixed tissue sections (4% formaldehyde) were incubated with sirius red solution for one hour at room temperature and rinsed in 0.01 N HCl to remove unbound dye.

Troponin T: Tissue sections were fixed in 4% phosphate-buffered formalin and stained with a mouse monoclonal antibody against human Troponin T (TnT) (clone 1C11, Abcam, UK) and visualized with an AlexaFluor^®^568-conjugated secondary antibody (Thermo Fischer Scientific, US). Nuclei were stained blue with DAPI.

Bright field and fluorescent images were then acquired using the Metafer Slide Scanning platform and stitched together using VSlide software (both supplied by Metasystem).

### On-chip permeabilization, reverse transcription and probe-release reactions

Tissue sections on oligonucleotide-covered glass slides were placed in ArrayIT mask holders to create reaction chambers for each tissue section, and incubated in 70 μl of a pre-permeabilization mixture [1x ExoI buffer (#B0293S, NEB), 0.2 μg/μl BSA (#B9000S, NEB)] at 37 °C for 30 minutes then washed in 100 μl 0.1x SSC (#S6639, Sigma-Aldrich) diluted in MQ RNase/DNase-free water. VN biopsy sections were then permeabilized by incubation in 70 μl of Permeabilization Mixture 1 [0.1% pepsin (#P7000-25G, Sigma-Aldrich), 0.1 M HCl (#318965-1000 ML, Sigma-Aldrich)] at 37 °C for 1 minute, and RAA biopsy sections by incubation in Permeabilization Mixture 2 [0.2% pepsin, 0.1 M HCl] at 37 °C for 15 minutes. Both types of sections were then washed in 100 μl 0.1x SSC.

Next, the tissue sections were incubated at 42 °C overnight (~17 hours) in reverse transcription mixture [1x First strand buffer (#18080-044, Invitrogen), 5 mM DTT (#18080-044, Invitrogen), 0.5 mM of each dNTP (#R0192, Fisher Scientific), 0.2 μg/μl BSA, 50 ng/μl Actinomyocin D (#A1410-2MG, Sigma-Aldrich), 1% DMSO (#472301-500 ML, Sigma-Aldrich), 20 U/μl Superscript III (#18080-044, Invitrogen) and 2 U/μl RNaseOUT (#10777-019, Invitrogen)]. Tissue was removed prior to the probe release in order to increase exposure of the RNA captured on-chip by incubation in 70 μl of Removal Mixture 1 [1:25 β-mercaptoethanol (#444203, CALBIOCHEM), RLT buffer (#79216, Qiagen)] at 56 °C for 1.5 hours with continuous shaking, washing in 100 μl 0.1x SSC, then incubation in 70 μl of Removal Mixture 2 [Proteinase K (#19131, Qiagen), PKD buffer (#1034963, Qiagen, pH7.5)] at 56 °C for 1 hour. After this tissue removal treatment, glass slides were washed in 2x SSC containing 0.1% SDS (#71736-100 ML, Sigma-Aldrich), at 50 °C for 10 minutes, followed by 1 min washes in 0.2x SSC and 0.1x SSC at room temperature. Finally, to detach the surface probes, 70 μl of a release mixture [1.08x Second strand buffer (#10812-014, Invitrogen), 8.75 μM of each dNTP, 0.2 μg/μl BSA, 0.1 U/μl USER enzyme (#M5505, NEB)] was added and the preparations were incubated at 37 °C for 2 hours.

### Preparation and sequencing of cDNA libraries

65 μl of the reaction mixture containing the released probes was collected and 2^nd^ strand synthesis, cDNA purification, *in vitro* transcription, aRNA purification, adapter ligation, post-ligation purification, a second 2^nd^ strand synthesis and purification were done using an automated MBS 8000 + system^22^. To determine the number of PCR cycles needed for indexing, 2 μl of the purified cDNA was mixed with 8 μl of a qPCR mixture [1.25x KAPA HiFi HotStart Readymix (#KK2601, KAPA Biosystems), 0.625 μM PCR lnPE1.0 primer (Eurofins), 12.5 nM PCR lnPE2.0 primer (Eurofins), 0.625 μM PCR Index primer (Eurofins), 1.25xEVA green (#31000, Biotium)]. Then qPCR amplifications were run using an qPCR instrument (Bio-Rad) and the following temperature program: 98 °C for 3 minutes, followed by 25 cycles of 98 °C for 20 s, 60 °C for 30 s and 72 °C for 30 s. The remaining purified cDNA was indexed and amplified using the following program: 98 °C for 3 minutes, followed by the optimal number of cycles (determined from the qPCR) of 98 °C for 20 s, 60 °C for 30 s and 72 °C for 30 s, finishing with a 5-min extension step at 72 °C and storage at 4 °C. The indexed library was purified using an automated MBS robot system^23^. Average lengths of the indexed libraries were assessed using a 2100 Bioanalyzer (Agilent) and concentrations were measured using a Qubit dsDNA HS Assay Kit (#Q32854, Life Technologies), according to the manufacturer’s instructions. Indexed libraries were diluted to 4 nM and subjected to paired-end sequencing using the Illumina NextSeq platform, according to the manufacturer’s instructions. Thirty-one bases from each forward read were used to determine the spatial barcode and 121 bases from the reverse read to cover the genetic region. Between 32 and 39 million raw read-pairs were generated from each LV sample and 55–64 million raw read-pairs from each RAA sample. None of the sequencing libraries reached saturation level.

### Quality control assay experiments

Quality control experiments were carried out with some modifications to the procedure mentioned in the *Permeabilization*, *reverse transcription and probe-release reactions performed on-chip* section, without the detachment of surface probes step. The reverse transcription mixture contained the same reagents as above except there was 0.5 mM of each dATP/dGTP/dTTP, 12.5 µM dCTP and 25 µM Cy3-dCTP (#NEL576001EA, PerkinElmer). Variations included as part of the optimization procedure included use of 5–10 μm thick sections, 0.1–0.2% pepsin concentrations and permeabilization times ranging between 1 and 15 minutes. Following all of the treatments the glass slides were scanned in a NimbleGen Microarray scanner (Roche) and signal intensities were measured using GenePix Pro 5.0 Microarray Acquisition & Analysis software.

### Image alignment

After probes had been released from a glass slide, it was incubated with 70 μl of a hybridization solution [0.96x PBS, 0.2 μM Cy3 anti-A probe (Eurofins), 0.2 μM Cy3 anti-frame probe (Eurofins)] at room temperature for 10 minutes. The slide was then washed in 2x SSC containing 0.1% SDS at 50 °C for 10 minutes, followed by 1-minute washes in 0.2x SSC and 0.1x SSC at room temperature. Fluorescent images were acquired using the Metafer Slide Scanning platform and stitched together using VSlide software. Both bright field and fluorescent images were manually aligned using Photoshop CS6 (Adobe).

### Total RNA extraction

Cardiac tissue was placed in Lysing Matrix D tubes (#116913050, MP Biomedicals) and homogenized in a FastPrep instrument (MP Biomedicals). Total RNA was extracted using a RNeasy Fibrous Tissue Mini Kit (#74704, Qiagen), according to the manufacturer’s instructions. The RNA integrity number (RIN) for each sample was assessed using a 2100 Bioanalyzer (Agilent).

### Sequence alignment and annotation

Removal of homopolymer stretches of 15 bases or more from reverse reads was followed by BWA-based quality trimming. Remaining reads shorter than 28 bases were discarded. The reverse reads were then mapped against a human genome rRNA contamination filter and those with apparent contaminants were discarded. Non-contaminated reads were then mapped against the human genome (Ensembl version GRCh38) using STAR^24^ (version 2.4.2a) with default settings. Mapped reads were quantified using htseq-count^25^ (version 0.6.1, mode: intersection-nonempty) with Ensembl gene annotations (version GRCh38.79) and non-annotated mapped reads were discarded. Each mapped annotated read was then de-multiplexed^26^ with its corresponding forward read (containing the spatial barcode) and all reads whose barcode did not match the reference barcode file were discarded. Duplicates were removed using information from the unique molecular identifiers (UMIs). More details about the analysis pipeline (version 0.5.6) can be found at https://github.com/SpatialTranscriptomicsResearch/st_pipeline.

### Comparing datasets

All scatter-, PCA-, volcano-, histo- and barplots presented here were generated in R (version 3.2.2) using its in-built functions if not otherwise specified. Read counts were normalized by DESeq. 2^27^, followed by adding a pseudocount of 1 and log-transformation.

Samples were compared pair-wise by first aggregating all counts from all barcodes. Genes not expressed in either of the two samples compared were removed before normalization, then Pearson correlation coefficients were calculated.

Complexity curves were generated by drawing 1, 10, 20 and 30 million subsamples of the raw fastq files from each dataset using seqtk^28^ (version 1.0), before sequence alignment and annotation as described in the *Sequence alignment and annotation* section. Reads from features within the dataset were extracted and counts from each barcode were aggregated.

Principal Component Analysis (PCA) was applied to aggregated counts from features within the dataset, using the prcomp function in R and data pertaining to the 1,000 most highly expressed genes after normalization.

Heatmap showing differences in gene expression between features in samples from individual 1 was generated solely using features showing expression of at least 500 genes. After normalization, data pertaining to the 1,000 genes with the highest variance across features were plotted using the Heatmap.2 function in the gplot package^29^ (version 2.17.0). Volcano plots of DE genes were generated using normalized aggregated counts from all barcodes. Genes showing more than a |log2fold change| > 1 and an adjusted p-value < 0.01 were considered to be differentially expressed.

Barplots showing numbers of unique transcripts and genes found within features were generated by summing transcripts and gene counts from each barcode.

Barplots illustrating comparison between ST data and bulk treated ST data shows the average number of relative counts per feature, after adding a pseudocount of 1 and log-transformation. The number of features that the relative counts are based on correspond to either features expressing the fetal gene (ST data) or to the total amount of features within the dataset (bulk treated ST data).

Heatmaps showing spatial expression patterns of four selected genes across tissue sections were generated from read counts in features across the dataset with at least 100 genes present, using the X and Y coordinates obtained from the barcode information.

### Spatial gene expression analysis and visualization

The dimensionality of data acquired from features within the dataset expressing at least 100 genes was reduced by *t*-SNE after normalization using DESeq. 2. Each feature was assigned a color representing its relationship to the other features analyzed using the same setup, with similar colors reflecting similar gene expression patterns.

### Oligonucleotide sequences

Surface reverse transcription oligonucleotide for quality control experiments: [AmC6]UUUUUGACTCGTAATACGACTCACTATAGGGACACGACGCTCTTCCGATCTNNNNNNNNTTTTTTTTTTTTTTTTTTTTVN

Surface reverse transcription oligonucleotides with spatial barcodes:

[AmC6]UUUUUGACTCGTAATACGACTCACTATAGGGACACGACGCTCTTCCGATCT[18mer_Spatial_Barcode_1to1007]WSNNWSNNVTTTTTTTTTTTTTTTTTTTTVN

Surface frame oligonucleotide:

[AmC6]AAATTTCGTCTGCTATCGCGCTTCTGTACC

aRNA ligation adapter:

[rApp]AGATCGGAAGAGCACACGTCTGAACTCCAGTCAC[ddC]

Second reverse transcription primer:

GTGACTGGAGTTCAGACGTGTGCTCTTCCGA

PCR primer InPE1.0 primer:

AATGATACGGCGACCACCGAGATCTACACTCTTTCCCTACACGACGCTCTTCCGATCT

PCR primer InPE2.0 primer:

GTGACTGGAGTTCAGACGTGTGCTCTTCCGATCT

PCR Index primer:

CAAGCAGAAGACGGCATACGAGATXXXXXXGTGACTGGAGTTC

Cy3 anti-A probe:

[Cy3]AGATCGGAAGAGCGTCGTGT

Cy3 anti-frame probe:

[Cy3]GGTACAGAAGCGCGATAGCAG

## Electronic supplementary material

Supplementary Material

## References

[1] Roger VL (2013). Epidemiology of heart failure. Circ. Res..

[2] Chowdhury A (2014). Expression of fibulin-6 in failing hearts and its role for cardiac fibroblastmigration. Cardiovasc. Res..

[3] Mearini G (2010). Atrogin-1 and MuRF1 regulate cardiac MyBP-C levels via different mechanisms. Cardiovasc. Res..

[4] Karamanlidis G (2013). Mitochondrial complex I deficiency increases protein acetylation and accelerates heart failure. Cell Metab..

[5] Akat KM (2014). Comparative RNA-sequencing analysis of myocardial and circulating small RNAs in human heart failure and their utility as biomarkers. Proc. Natl. Acad. Sci. USA.

[6] Yang KC (2014). Deep RNA sequencing reveals dynamic regulation of myocardial noncoding RNAs in failing human heart and remodeling with mechanical circulatory support. Circulation.

[7] Ståhl PL, Salmén F (2016). Visualization and analysis of gene expression in tissue sections by spatial transcriptomics. Science.

[8] Lonsdale J (2013). The Genotype-Tissue Expression (GTEx) project. Nat. Genet..

[9] Gaborit B (2015). Human epicardial adipose tissue has a specific transcriptomic signature depending on its anatomical peri-atrial, peri-ventricular, or peri-coronary location. Cardiovasc. Res..

[10] Braas K (1992). Expression of peptidylglycine alpha-amidating monooxygenase: an *in situ* hybridization and immunocytochemical study. Endocrinology.

[11] Bodine SC (2001). Identification of ubiquitin ligases required for skeletal muscle atrophy. Science.

[12] Zidar, N. *et al*. Cyclooxygenase in normal human tissues - is COX-1 really a constitutive isoform, and COX-2 an inducible isoform? *J*. *Cell*. *Mol*. *Med*. 10.1111/j.1582-4934.2008.00430.x (2009). 10.1111/j.1582-4934.2008.00430.x PMC4516524 18657230

[13] Jain R (2015). Integration of Bmp and Wnt signaling by Hopx specifies commitment of cardiomyoblasts. Science.

[14] Dirkx E, da Costa Martins PA, De Windt LJ (2013). Regulation of fetal gene expression in heart failure. Biochim. Biophys. Acta - Mol. Basis Dis..

[15] Rajabi M, Kassiotis C, Razeghi P, Taegtmeyer H (2007). Return to the fetal gene program protects the stressed heart: A strong hypothesis. Heart Fail. Rev..

[16] Taegtmeyer H, Sen S, Vela D (2010). Return to the fetal gene program: A suggested metabolic link to gene expression in the heart. Ann. N. Y. Acad. Sci..

[17] Coles JG (2005). Cardioprotective stress response in the human fetal heart. J. Thorac. Cardiovasc. Surg..

[18] Bedada FB (2014). Acquisition of a quantitative, stoichiometrically conserved ratiometric marker of maturation status in stem cell-derived cardiac myocytes. Stem Cell Reports.

[19] Ahuja P (2013). Divergent mitochondrial biogenesis responses in human cardiomyopathy. Circulation.

[20] Linde, C. *et al*. Rationale and design of the PREFERS (Preserved and Reduced Ejection Fraction Epidemiological Regional Study) Stockholm heart failure study: an epidemiological regional study in Stockholm county of 2. 1 million inhabitants. *Eur*. *J*. *Heart Fail*. 1–11, 10.1002/ejhf.599 (2016). 10.1002/ejhf.599 27384611

[21] Vickovic S (2016). Massive and parallel expression profiling using microarrayed single-cell sequencing. Nat. Commun..

[22] Jemt, A. *et al*. An automated approach to prepare tissue-derived spatially barcoded RNA-sequencing libraries. *Sci*. *Rep*. (2016). 10.1038/srep37137 PMC5111054 27849009

[23] Lundin S (2010). Increased throughput by parallelization of library preparation for massive sequencing. PLoS One.

[24] Dobin A (2013). STAR: ultrafast universal RNA-seq aligne≥r. Bioinformatics.

[25] Anders S, Pyl PT, Huber W (2014). HTSeq - A Python framework to work with high-throughput sequencing data. Bioinformatics.

[26] Sjöstrand, J. & Fernandez Navarro, J. TagGD. https://github.com/JoelSjostrand/taggd

[27] Love MI, Huber W, Anders S (2014). Moderated estimation of fold change and dispersion for RNA-seq data with DESeq. 2. Genome Biol..

[28] Li, H. seqtk. https://github.com/lh3/seqtk (2013).

[29] Warnes, G. R. *et al*. gplots: Various R programming tools for plotting data. *R Packag*. *version* **2** (2009).

