# Supplementary material for: Spatial detection of fetal marker genes expressed at low level in adult human heart tissue
Source: Sci Rep. 2017 Oct 11;7:12941. doi: 10.1038/s41598-017-13462-5 (PMC5636908; doi:10.1038/s41598-017-13462-5)
Supplement: Supplementary file 1 — Supplementary Material [file 41598_2017_13462_MOESM1_ESM.pdf]

Supplementary Material for

Spatial detection fetal marker genes expressed at low level in adult human heart tissue

Michaela Asp[1], Fredrik Salmén[1†], Patrik L. Ståhl[2†], Sanja Vickovic[1†], Ulrika Felldin[3], Marie Löfling[3], José Fernandez Navarro[2], Jonas Maaskola[1], Maria J. Eriksson[3,4], Bengt Persson[5,6], Matthias Corbascio[7], Hans Persson[8,9], Cecilia Linde[10,11], Joakim Lundeberg[1*]

*Correspondence to Joakim Lundeberg; joakim.lundeberg@scilifelab.se

This PDF file includes:

Supplementary figures

Supplementary tables

Supplementary Figure 1.

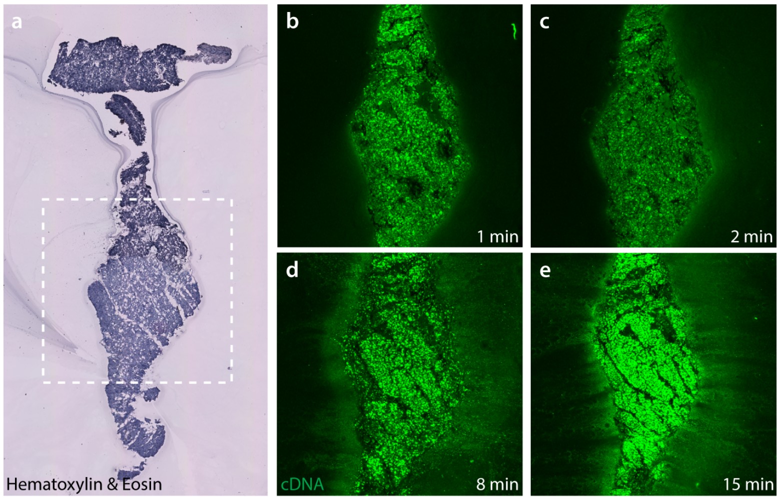

Supplementary Figure 2.

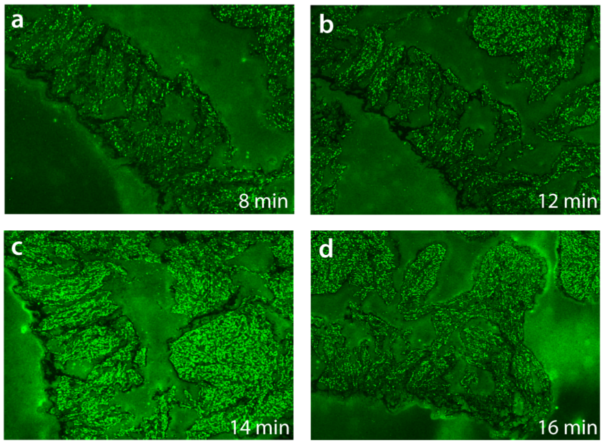

Supplementary Figure 3

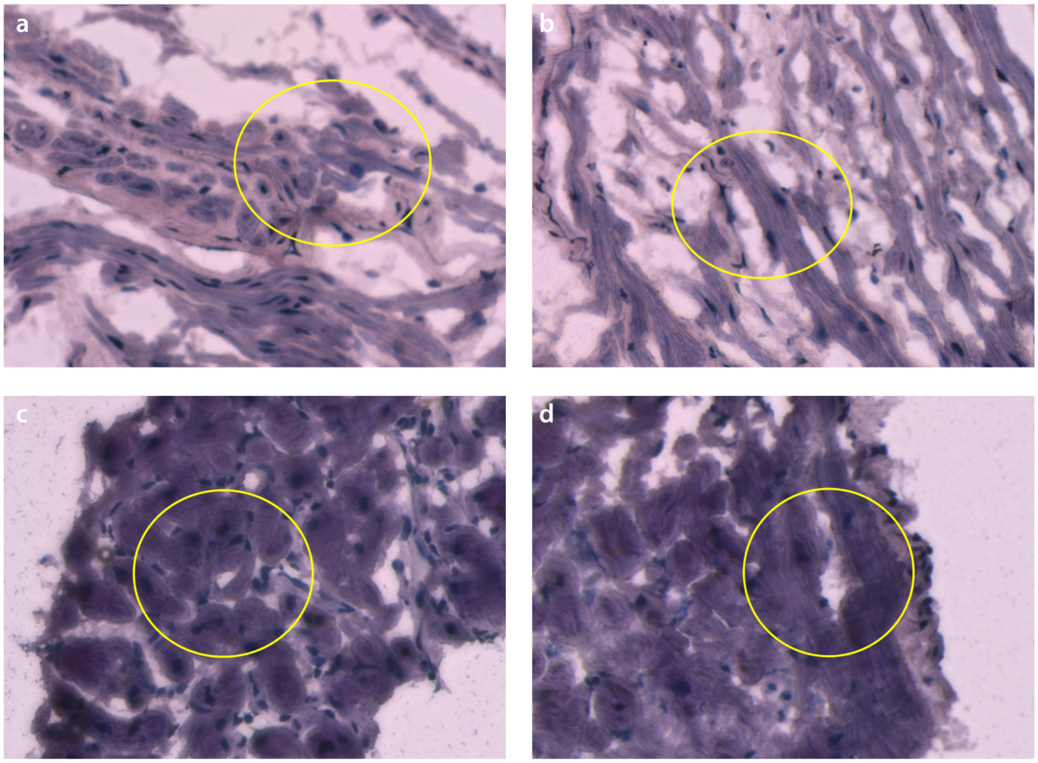

Supplementary Figure 4.

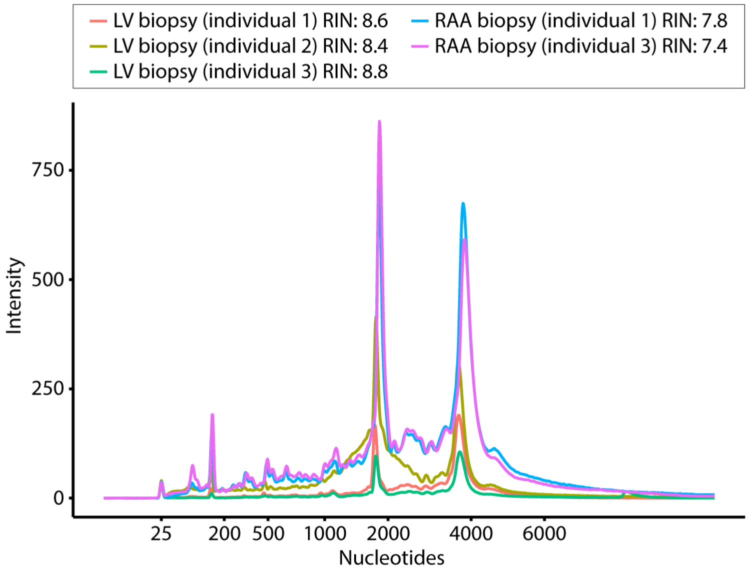

Supplementary Figure 5.

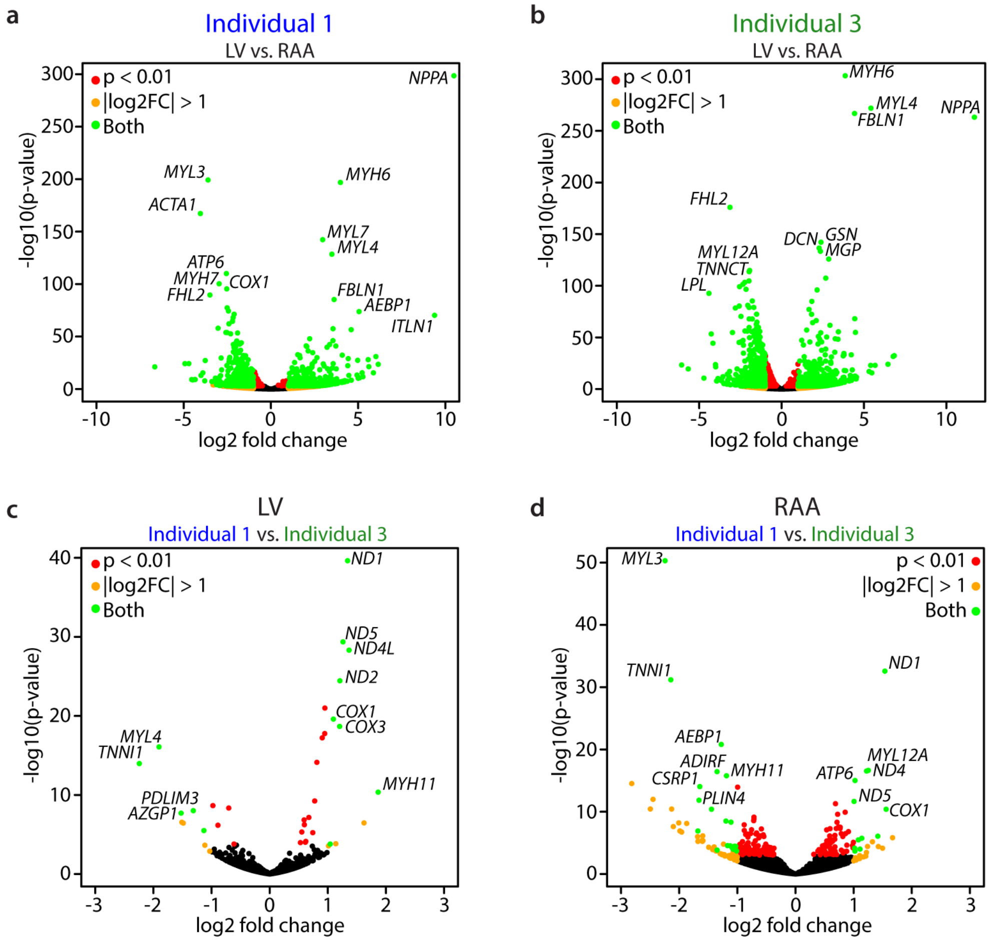

Supplementary Figure 6.

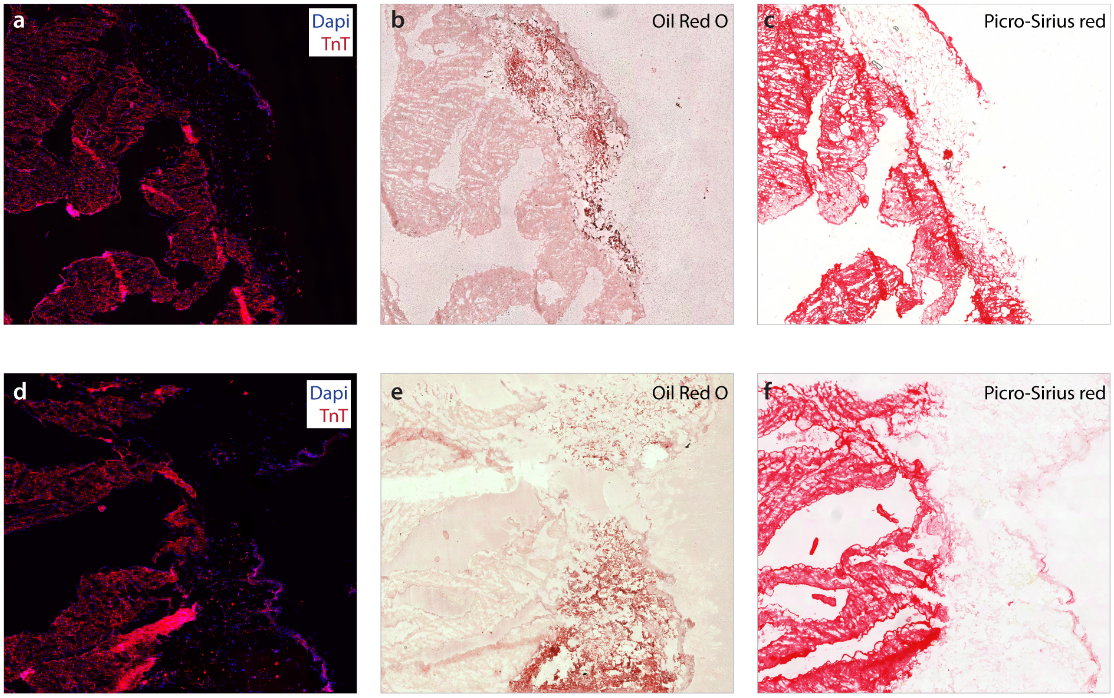

Supplementary Figure 7.

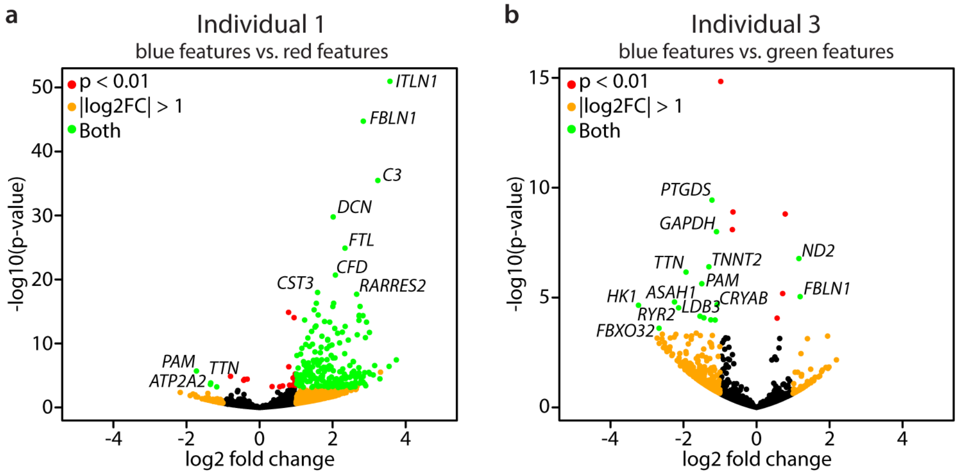

Supplementary Table 1.

	NKX2.5	GATA4	TBX5	TBX20	SSEA-1	HOPX
**LV Individual 1**	70/348379 121 features	9/348379 121 features	8/348379 121 features	0/348379 121 features	0/348379 121 features	2/348379 121 features
**RAA Individual 1**	207/1240352 574 features	77/1240352 574 features	119/1240352 574 features	6/1240352 574 features	6/1240352 574 features	21/1240352 574 features
**LV Individual 2**	30/351861 107 features	16/351861 107 features	6/351861 107 features	0/351861 107 features	1/351861 107 features	1/351861 107 features
**LV Individual 3**	47/364420 192 features	17/364420 192 features	10/364420 192 features	0/364420 192 features	0/364420 192 features	2/364420 192 features
**RAA Individual 3**	241/1817145 899 features	86/1817145 899 features	145/1817145 899 features	0/1817145 899 features	18/1817145 899 features	33/1817145 899 features

Supplementary Table 2.

	NKX2.5	GATA4	TBX5	TBX20	SSEA-1	HOPX
**LV Individual 1**	70/166926 48 features	9/25262 7 features	8/30695 8 features	0/0 0 features	0/0 0 features	2/9472 2 features
**RAA Individual 1**	207/363226 155 features	77/135571 62 features	119/231334 97 features	6/2880 4 features	6/16940 6 features	21/43163 20 features
**LV Individual 2**	30/74876 26 features	16/38437 13 features	6/11786 6 features	0/0 0 features	1/1045 1 feature	1/2762 1 feature
**LV Individual 3**	47/97531 33 features	17/37930 13 features	10/26111 7 features	0/0 0 features	0/0 0 features	2/3445 1 feature
**RAA Individual 3**	241/467853 198 features	86/161717 73 features	145/288333 127 features	0/0 0 features	18/31980 18 features3	33/82292 32 features

Supplementary Figure 8.

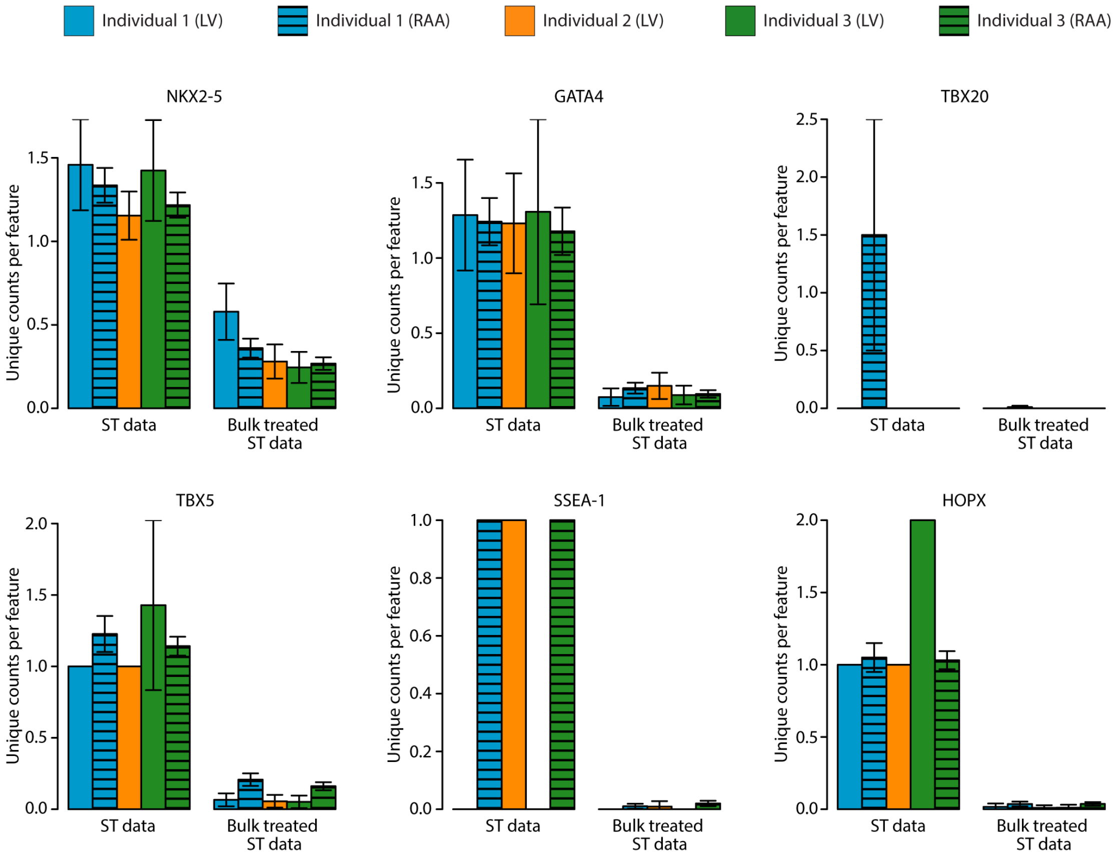

Supplementary Figure 9.

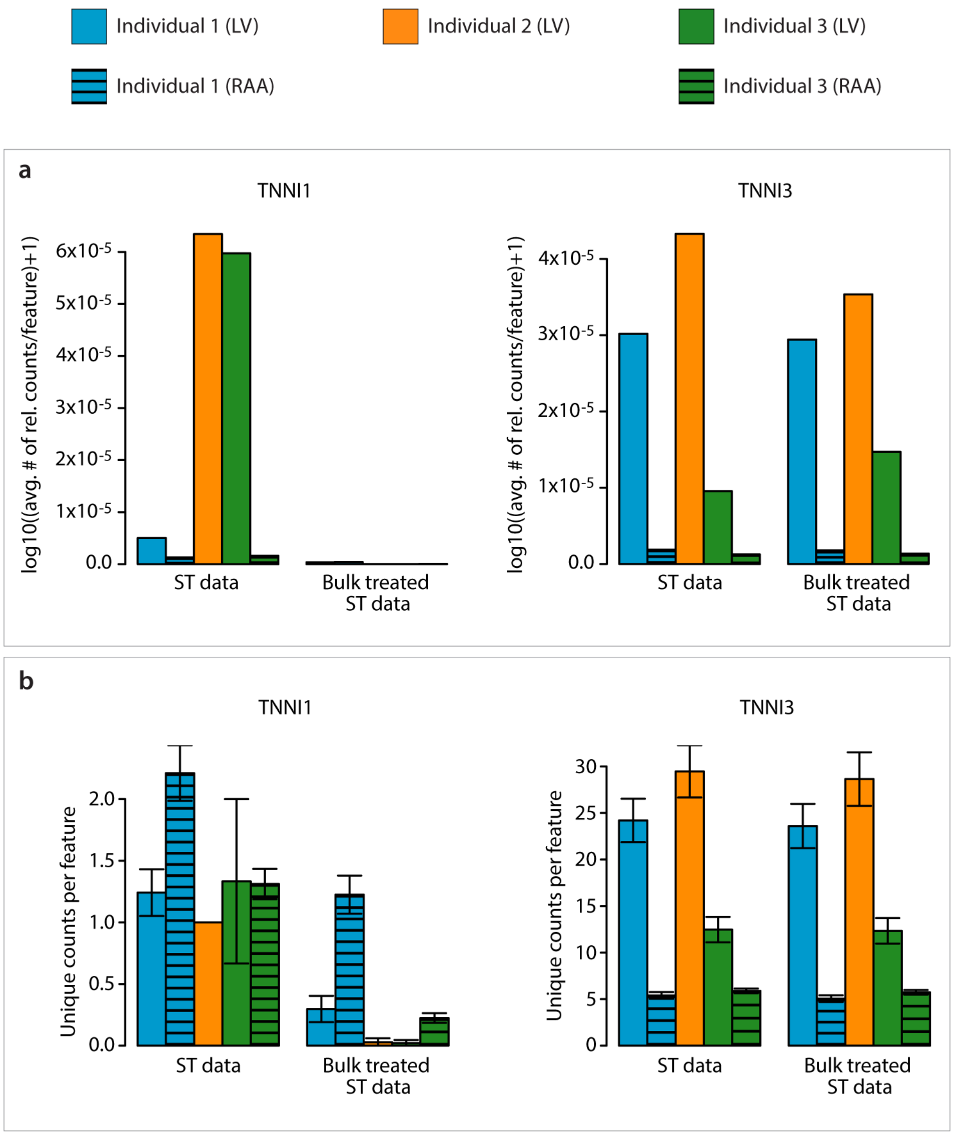

Supplementary Table 3.

	TNNI1	TNNI3
**LV Individual 1**	36/348379<br>121 features	2856/348379<br>121 features
**RAA Individual 1**	703/1240352<br>574 features	2903/1240352<br>574 features
**LV Individual 2**	3/351861<br>107 features	3065/351861<br>107 features
**LV Individual 3**	4/364420<br>192 features	2370/364420<br>192 features
**RAA Individual 3**	202/1817145<br>899 features	5153/1817145<br>899 features

Supplementary Table 4.

	TNNI1	TNNI3
**LV**<br>**Individual 1**	36/107606<br>29 features	2856/348376<br>118 features
**RAA**<br>**Individual 1**	703/741278<br>318 features	2903/1235889<br>538 features
**LV**<br>**Individual 2**	3/6845<br>3 features	3065/295680<br>104 features
**LV**<br>**Individual 3**	4/9691<br>3 features	2370/566356<br>190 features
**RAA**<br>**Individual 3**	202/357448<br>154 features	5153/2050541<br>875 features

Figure legends.

Supplementary Figure 1.

Illustrative results of quality control assay of consecutive tissue sections from a right ventricle needle biopsy.

(**a**) Hematoxylin- and eosin-stained tissue section, the marked area corresponds to the area shown in panels b-e. (**b-e**) Cy-3 signals from 10 µm tissue sections treated with 0.1% (w/v) pepsin for indicated times showing increase in amounts of transcripts diffusing from the tissue with time. Intensities of signals from sections shown in panels c, d and e are 0.63-, 1.25- and 1.5-fold stronger than those from the section shown in panel b. Samples derive from the same experiment and images were processed in parallel using the GenePix Pro 5.0 software.

Supplementary Figure 2.

Illustrative results of quality control assay of RAA tissue sections.

(**a-d**) Cy-3 signals from 5 µm tissue sections treated with 0.2% pepsin (w/v), for indicated times: intensities of signals from sections shown in panels b, c and d are 2-, 5- and 2.6-fold stronger than those from the section shown in panel a. Samples derive from the same experiment and images were processed in parallel using the GenePix Pro 5.0 software.

Supplementary Figure 3

Magnified images of cardiac tissue sections and the corresponding feature position at that location.

(**a-d**) Hematoxylin and eosin stained cardiac tissue sections where the yellow circles indicate the position of the underlying feature. (**a, b**) Features covering a cross-section and longitudinal section of cardiomyocytes from the RAA. (**c, d**) Features covering a cross-section and longitudinal section of cardiomyocytes from the LV.

Supplementary Figure 4.

RNA quality of adult cardiac biopsies included in this study.

Supplementary Figure 5.

Variations in gene expression within and between individuals.

(**a-d**) Volcano plots showing differential gene expression between LV and RAA tissue sections (a, b), and tissue sections from the same cardiac region but different individuals (c, d). Numbers of genes with a |log2fold change| > 1 and an adjusted p-value < 0.01 in the comparisons shown in panels a, b, c and d are 911, 1061, 13 and 31, respectively.

Supplementary Figure 6.

Histological staining of RAA tissue sections.

Consecutive tissue sections of RAA from individual 1 (**a-c**) and individual 3 (**d-f**). Tropin T (TnT), Oil Red O and Picro-Sirius red show the spatial location of cardiomyocytes, adipose tissue and collagen (extracellular matrix).

Supplementary Figure 7.

Differential gene expression within RAA tissue sections.

(**a**) Volcano plot showing DE genes between the blue and red areas in Fig. 3b. Numbers of genes with a |log2fold change| > 1 and an adjusted p-value < 0.01 in blue and red areas are 4 and 212, respectively. (**b**) Volcano plot showing DE genes between the blue and green areas in Fig. 3f. Data for the COX1 gene is not shown here as it had a substantially lower p-value ($2.8x10^{-99}$) than all other genes. Numbers of genes with a |log2fold change| > 1 and adjusted p-value < 0.01 in blue and green areas are 14 and 2, respectively.

Supplementary Table 1.

Bulk treated ST data.

Here, unique read counts are aggregated from features of two replicates. Upper panel: Unique read counts of the indicated fetal gene/Total number of unique read counts. Lower panel: Number of features within the two replicates.

Supplementary Table 2.

ST data.

Here, unique read counts are based only from features of two replicates expressing the indicated fetal gene. Upper panel: Unique read counts of the indicated fetal gene/Total number of unique read counts from features expressing the indicated fetal gene. Lower panel: Number of features within the two replicates that express the fetal gene.

Supplementary Figure 8.

Number of fetal gene read counts per feature.

Barplots showing unique fetal gene read counts per feature. In the ST data, the number of features is based on features displaying read counts from indicated fetal marker gene. In the bulk treated ST data, the number of features is based on the total amount of features within the dataset. Error bars indicate standard errors.

Supplementary Figure 9.

Detection of TNNI1 and TNNI3 expression within adult heart tissue sections.

(**a**) Barplots showing the average number of relative fetal gene counts per feature, as described in Fig. 4. (**b**) Barplots showing unique TNNI1 and TNNI3 read counts per feature, as described in Supplementary Fig. 8. Error bars indicate standard errors.

Supplementary Table 3.

Bulk treated ST data.

Here, unique read counts are aggregated from features of two replicates. Upper panel: Unique read counts of the indicated gene (TNNI1 or TNNI3)/Total number of unique read counts. Lower panel: Number of features within the two replicates.

Supplementary Table 4.

ST data.

Here, unique read counts are based only from features of two replicates expressing the indicated gene (TNNI1 or TNNI3). Upper panel: Unique read counts of the indicated gene/Total number of unique read counts from features expressing the indicated gene. Lower panel: Number of features within the two replicates that express either TNNI1 or TNNI3.
